# Verticillium Suppression Is Associated with the Glucosinolate Composition of *Arabidopsis thaliana* Leaves

**DOI:** 10.1371/journal.pone.0071877

**Published:** 2013-09-05

**Authors:** Katja Witzel, Franziska S. Hanschen, Monika Schreiner, Angelika Krumbein, Silke Ruppel, Rita Grosch

**Affiliations:** 1 Department of Plant Nutrition, Leibniz-Institute of Vegetable and Ornamental Crops, Grossbeeren, Germany; 2 Department of Plant Quality, Leibniz-Institute of Vegetable and Ornamental Crops, Grossbeeren, Germany; 3 Department of Plant Health, Leibniz-Institute of Vegetable and Ornamental Crops, Grossbeeren, Germany; Centro de Investigación y de Estudios Avanzados, Mexico

## Abstract

The soil-borne fungal pathogen *Verticillium longisporum* is able to penetrate the root of a number of plant species and spread systemically via the xylem. Fumigation of Verticillium contaminated soil with Brassica green manure is used as an environmentally friendly method for crop protection. Here we present a study focused on the potential role of glucosinolates and their breakdown products of the model plant *Arabidopsis thaliana* in suppressing growth of *V. longisporum*. For this purpose we analysed the glucosinolate composition of the leaves and roots of a set of 19 key accessions of *A. thaliana*. The effect of volatile glucosinolate hydrolysis products on the *in vitro* growth of the pathogen was tested by exposing the fungus to hydrated lyophilized plant tissue. Volatiles released from leaf tissue were more effective than from root tissue in suppressing mycelial growth of *V. longisporum*. The accessions varied in their efficacy, with the most effective suppressing mycelial growth by 90%. An analysis of glucosinolate profiles and their enzymatic degradation products revealed a correlation between fungal growth inhibition and the concentration of alkenyl glucosinolates, particularly 2-propenyl (2Prop) glucosinolate, respectively its hydrolysis products. Exposure of the fungus to purified 2Prop glucosinolate revealed that its suppressive activity was correlated with its concentration. Spiking of 2Prop glucosinolate to leaf material of one of the least effective *A. thaliana* accessions led to fungal growth suppression. It is suggested that much of the inhibitory effect observed for the tested accessions can be explained by the accumulation of 2Prop glucosinolate.

## Introduction

The soil-borne fungi *Verticillium longisporum* and *V. dahliae*, responsible for vascular diseases, are both damaging with respect to the yield and quality of a number of economically important crops worldwide [1], [2]. They typically infect their hosts by root penetration via the cortex and endodermis in response to plant exudates, and subsequently spread systemically via xylem in the form of conidia in the upper part of plant [3]. Infections by these pathogens affect water and nutrition transport in the plants with the consequences of typical symptoms such as wilting, stunting and chlorosis [4]. Within the plant, *Verticillium* spp. secretes various polysaccharide lyases able to degrade the host's primary cell wall and enable proliferation in the xylem [5]. The host response to infection has been widely characterized at both the transcript [6], [7], [8], [9], [10], [11] and the protein [12], [13], [14] levels. In tomato [15] and cotton [16] the presence of the immune receptor Ve confers resistance, and the corresponding virulence effector has been described in the pathogen [17]. A wide range of responses to Verticillium infection has been observed among *Arabidopsis thaliana* accessions, and genes implicated in phytohormone signalling [18] and development [19], [20] make a contribution to this variation.

Plants have evolved inducible and preformed defence mechanisms to counteract pathogen attacks. Production of secondary metabolites with antimicrobial properties is a preformed defence mechanism. A known group of constitutive natural plant compounds are glucosinolates found mainly in Capparales and almost exclusively in *Brassicaceae* family including economically important crops as well as in the model plant *A. thaliana* [21]. Nitrogen- and sufur-containing glucosinolates, derived from chain elongated and glucosidated amino acids, represent a diverse set of secondary metabolites [22]. In their intact form, they appear to be relatively inactive, but upon hydrolysis, they display a range of herbivore- and pathogen-suppressing activity [23], [24]. Their degradation is catalysed by myrosinase and is regulated by proteins which control the synthesis of isothiocyanates (ITCs), nitriles and thiocyanates, among others [25]. The unstable aglycone that is produced upon glucosinolate degradation by myrosinase is converted into ITC by default. However, the nature of the hydrolysis products is mainly defined by the structure of the glucosinolate side chain and depends on the plant species [21]. In Arabidopsis, depending on the glucosinolate side chain, hydrolysis conditions, and presence of specific protein factors, the formation of nitriles and epithionitriles can be favoured. This shift is controlled by nitrile-specifier proteins [26], epithiospecifier proteins ESP [27], thiocyanate-forming proteins [28] and epithiospecifier modifier proteins [29]. The anti-fungal activity of oils purified from mustard was discovered as early as the 1930s [30], and a wealth of data has since confirmed these early findings [31]. While some of their fungicidal activity has been ascribed to non-volatile degradation products, most of it derives from volatile products, including 2-propenyl ITC (2Prop-ITC), 3-butenyl ITC (3But-ITC) and benzyl ITC [31].

The antifungal activity of volatile glucosinolate breakdown products, mainly ITCs, are assumed as driving compounds in biofumigation where crop residues (particularly those of *Brassica* spp.) with high glucosinolate content are incorporated into the soil for control of soil-borne pathogens [32], [33]. The hydrolysis of glucosinolates in the residue is an important component of this control, acting against fungi [34], [35], [36], [37], [38], [39], [40], bacteria [41], [42] and nematodes [43], [44]. Although it has been established that the severity of the disease caused by a number of pathogens can be notably attenuated when the host's glucosinolate composition is transgenically modified [45], there has been no systematic attempt until now to determine the extent to which genetic variation in glucosinolate composition affects pathogen growth within the plant. Here, we report an analysis of genetic variation with respect to the volatile glucosinolate breakdown product composition from the leaves and roots of *A. thaliana*. As a bioassay, we have measured the *in vitro* mycelial growth of *V. longisporum* in response to volatile emission upon tissue damage of leaves and roots. We hypothesize that the glucosinolate profile correlates with disease suppression of *V. longisporum* in a set of 19 key accessions of *A. thaliana* accessions.

## Experimental Procedures

### Cultivation of *Verticillium* spp

*A. thaliana* plants were inoculated with either one of two *V. longisporum* isolates 43-3 [46] or VD-1 [47] or *V. dahliae* isolate GU060637 (kindly provided by Valerie Grimault, GEVES, Angers, France). The fungi were cultivated at 25°C in the dark on potato dextrose agar (PDA) (VWR International GmbH, Germany). Conidial suspensions were prepared by inoculating 500 mL sucrose sodium nitrate medium with five mm diameter plugs excised from a PDA plate, and shaking the culture at room temperature for three weeks.

### Plant material, growth and inoculation method

The 19 *A. thaliana* (L.) Heynh. accessions investigated were Bur-0, Can-0, Col-0, Ct-1, Edi-0, Hi-0, Kn-0, Ler-0, Mt-0, No-0, Oy-0, Po-0, Rsch-4, Sf-2, Tsu-0, Wil-2, Ws-0, Wu-0 and Zu-0, which together make up the set of parents used by Kover *et al.* [48] to create a MAGIC (Multiparent Advanced Generation Intercross) population (kindly provided by L. Westphal, IBP Halle, Germany). All plants were grown in sand watered with nutrient solution, as described by Gibeaut *et al.* [49], and were exposed to an 8 h photoperiod provided by artificial lighting (300 µmol m^−2^ s^−1^) with a light temperature of 20°C and a dark temperature of 18°C. After five weeks, leaf and root tissue was harvested separately and lyophilized to provide the material both for glucosinolate analysis and the anti-fungal growth bioassay.

Two week old Bur-0 and Ler-0 plants were also inoculated with each of the *Verticillium* sp. isolates (or with water as a control). For this purpose, a conidial suspension was homogenised in a blender, filtered and adjusted to 10^6^ conidia mL^−1^. A 10 mL aliquot was poured over the surface of each pot, and the plants were cultivated for a further five weeks, before harvesting and lyophilizing their leaves and roots. This material was used to quantify the fungal DNA present in the plants' tissue. Reproducibility of results was confirmed in two independent experiments.

### Anti-fungal growth bioassay

Agar plugs (5 mm diameter) were removed from the margin of mycelial growth on a PDA culture of *V. longisporum* 43-3, transferred to a fresh PDA plate and held at 25°C in the dark for three days. After that, the plates were turned upside down with the fungus now positioned at the top. A sterile filter paper was placed in the lid, and 0.3 g of lyophilized plant tissue (leaf or root) was spread over its upper surface. Myrosinase-induced hydrolysis of the glucosinolate was initiated by moistening the filter paper with 1.8 mL sterile water, after which the plate was sealed. The diameter of the mycelial mat was measured after four days of incubation at 25°C, and compared to the mycelial growth on similarly treated plates where the plant material had not been included. Each of these experiments was represented by five technical replicates. Growth-suppressive effects of selected accessions Bur-0, Can-0, Edi-0, Hi-0, Ws-0 and Wu-0 was verified in two sets of independently grown plants.

In further experiments, a concentration range of 0 to 4 mg of purified 2Prop glucosinolate prepared from horseradish (sinigrin hydrate, obtained from Sigma-Aldrich Chemie GmbH, Germany) dissolved in sterile filtrated 0.03 M citrate buffer, pH 6.5, was applied to the filter instead of the plant material. Hydrolysis was initiated by the addition of 0.1 U thioglucosidase extracted from white mustard (Sigma-Aldrich Chemie GmbH, Germany). Mycelial growth was assessed after four days of incubation on five technical replicates per treatment. This experiment was performed twice to ensure reproducibility.

Spiking experiments using one of the least effective *A. thaliana* accession Oy-0 were performed as described above, except that 0 to 4 mg of purified 2Prop glucosinolate was dissolved in sterile water and added to 0.3 g of lyophilized leaf material. Hydrolysis of 2Prop glucosinolate was initiated by the plants endogenous myrosinase. Mycelial growth was assessed after four days of incubation on five technical replicates per treatment and compared to plates where plant material had not been included. This experiment was performed twice to ensure reproducibility.

### Glucosinolate analysis

Desulfo-glucosinolate profiles and concentrations were derived using a modified HPLC protocol [50]. Duplicates of lyophilized plant tissue (20 mg) were heated to 75°C and held there for 1 min, and then extracted by the addition of 0.75 mL 70% methanol. After incubating at 75°C for 10 min, the extracts were centrifuged for 5 min, the supernatant removed, and the residue re-extracted twice more in 0.5 mL 70% methanol at 70°C. To convert the combined extracts to desulfo-glucosinolates, extracts were loaded on a 500 µL DEAE-Sephadex A-25 ion-exchanger (Sigma-Aldrich Chemie GmbH, Germany). Prior to sample loading, the column was first equilibrated in 2 M acetic acid, then pre-treated by the addition of two 1 mL aliquots of 6 M imidazole-formate (Carl Roth GmbH, Germany) in 30% v/v formic acid, followed by two washes with 1 mL deionized water. The column was washed twice with 1 mL 20 mM sodium acetate buffer pH 4.0 (Sigma-Aldrich Chemie GmbH, Germany), and 75 µL purified *Helix pomatia* aryl sulfatase (Roche Diagnostics GmbH, Germany) was loaded and left to stand for 12 h. Desulfo compounds were eluted with 1 mL deionized water. Desulfo-glucosinolate quantification was carried out by HPLC (Merck HPLC pump L-7100, DAD detector L-7455, automatic sampler AS-7200 and HPLC Manager-Software D-7000) using a Spherisorb ODS2 column (Bischoff, Germany, 3 µm, 125×4 mm). The separation employed a 0–20% v/v aqueous acetonitrile gradient from minutes 2–34, 20% v/v aqueous acetonitrile from minutes 35–40, and finally 100% acetonitrile from minutes 41–50, with a flow rate of 0.7 mL min^−1^. Detection was carried out at 229 nm. Glucosinolate concentrations were calculated using 2Prop glucosinolate as an external standard and the response factor of each compound relative to 2Prop glucosinolate. Where possible, desulfo-glucosinolates were identified following Zimmermann et al. [51], on the basis of protonated molecular ions [M+H]^+^ where the fragment ions corresponded to [M+H - glucose]^+^ by HPLC-ESI–MS^2^ using Agilent 1100 series (Agilent Technologies, Germany) operating in the positive ionization mode. Each determination was performed in duplicate. Hierarchical clustering of glucosinolate profiles was performed using MultiexperimentViewer MeV v4.7.4, based on Euclidean distance and average linkage clustering [52].

### Analysis of glucosinolate hydrolysis products derived from leaf tissue or purified 2Prop glucosinolate

For the determination of enzymatically formed breakdown products of the GSL, the method of Lambrix et al. (2001) was adapted. Either one mL of water was added to 50 mg of lyophilized plant tissue in centrifugal tubes and left for 30 min at room temperature for glucosinolate hydrolysis or 0.4 or 8 mg of purified 2Prop glucosinolate dissolved in the sterile filtrated citrate buffer described in 6.3 was hydrolysed for 2 h or 24 h by adding 0.1 U thioglucosidase. Next, 2 mL of methylene chloride (Carl Roth GmbH, Germany; GC Ultra Grade) and 100 µL of 2 mM benzonitrile in methylene chloride as internal standard (Sigma-Aldrich Chemie GmbH, Germany; ≥99.9%) were added and the tubes were sealed. After shaking for 20 sec and centrifugation for 5 min, the methylene chloride layer was removed and filtered through a small column of anhydrous sodium sulfate (VWR International GmbH, Germany; ≥99%) to remove residual water. The remaining aqueous layer was re-extracted with 2 mL of methylene chloride. The dried extracts were combined, concentrated under nitrogen gas flow to 300 µL and transferred into a vial. Samples were analyzed by gas chromatography-mass spectrometry detection (GC-MS) using an Agilent 6890 A Series GC System (Agilent Technologies, Germany) with a Gerstel Multi Purpose Sampler MPS2 (Gerstel GmbH & Co. KG, Germany) and an Agilent 5973 Network MSD. The GC was equipped with an Optima 5 MS column (Macherey-Nagel, Germany, 30 m×0.25 mm×0.25 µm film). After splitless injection of 1 µL of the sample at 190°C, analytes were separated, using helium as carrier gas (1.8 mL/min), and a temperature gradient starting at 35°C (3 min) and raising up to 50°C with 9°C/min. After holding this temperature for 7 min, the temperature increased to 230°C with 9°C/min and then with 35°C/min to 310°C. The temperature of the transfer line was 310°C, the ion source of the MSD was set to 230°C. Mass spectra were acquired in the EI mode (70 eV) in the full scan mode (TIC) for the plant tissue samples (*m/z* 30–350) or in the selected ion monitoring mode (SIM) for the hydrolysed 2Prop glucosinolate samples (Quantifier ions: *m/z* 41 for 2Prop-CN, *m/z* 99 for 2Prop-ITC and *m/z* 103 for the internal standard benzonitrile). Analytes were identified by comparing mass spectra and retention times with those of authentic standards and with literature data [53], [54]. Analyte content was calculated using benzonitrile as internal standard and the response factor (RF) of each compound relative to benzonitrile. The RF were experimentally determined for 2Prop-ITC (RF_TIC_ = 1.70, RF_SIM_ = 3.07), 3-butenenitrile (2-Prop-CN; RF_TIC_ = 3.70, RF_SIM_ = 7.32), 4-pentenenitrile (3But-CN; RF_TIC_ = 2.45), and 3-(methylthio)propyl ITC (3MTP-ITC; RF_TIC_ = 1.07) (all purchased from Sigma-Aldrich Chemie GmbH, Germany); 3-hydroxypropionitrile (RF_TIC_ = 7.67; Thermo Fischer Scientific, Belgium), 3But-ITC (RF_TIC_ = 1.06) and 4-pentenyl ITC (4-Pent-ITC; RF_TIC_ = 1.14) (both purchased from TCI Deutschland GmbH, Germany), 4-(methylthio)butyl ITC (4MTB-ITC; RF_TIC_ = 0.76; Santa Cruz Biotechnology, Germany), and for 4-(methylsulfinyl)butyl ITC (4MSOB-ITC; RF_TIC_ = 3.01; Enzo Life Sciences GmbH, Germany). For those compounds, that were commercially not available, the RF of the chemically most similar compound was used: For the epithionitriles of 2Prop and 3-But glucosinolate the RF of the corresponding ITC was used, diastereometric 3-hydroxy-4,5-epithiopentylnitrile (2OH3But-EPT) and 5-vinyl-1,3-oxazolidine-2-thione (OZT) were calculated with the RF of 3-But-ITC. The corresponding nitriles of 3-(methylthio)propyl (3-MTP) glucosinolate, 4-(methylthio)butyl (4-MTB) glucosinolate and 4-(methylsulfinyl)butyl (4MSOB) glucosinolate were calculated with the RF of the analogous ITC. The degradation products of 8-(methylthio)octyl (8MTO) glucosinolate were calculated with the RF of 4MTB-ITC and all sulfinyl nitriles and ITC were calculated with the RF of 4MSOB-ITC. For the quantification of degradation products of the 3-hydroxypropyl glucosinolate the RF determined for 3-hydroxypropionitrile was utilized. The limit of detection ranged between 0.9 µM (4Pent-ITC) and 15.5 µM (3-hydroxypropionitrile).

### DNA extraction and qRT-PCR analysis

Extraction of DNA from infected plant material was performed following Tinker et al. [55], with the inclusion of an additional DNA purification procedure [56]. The integrity and quantity of the DNA were assessed photometrically using a NanoDrop ND-1000 device (PeqLab GmbH, Germany).

The abundance of fungal DNA present in the plant material was estimated by a PCR based on the primer pair VDS1 (5′-CAC ATT CAG TTC AGG AGA CGG A-3′) and VDS2 (5′-CCT TCT ACT GGA GTA TTT CGG-3′), which specifically amplifies a 521 bp product from a template of either *V. dahliae* [57] or *V. longisporum* DNA. The amplicon was generated by imposing an initial denaturation of 95°C/3 min, followed by 40 cycles of 95°C/20 s, 66°C/20 s, 72°C/60 s. The template DNA was diluted tenfold in sterile water to an approximate concentration of 10 ng µL^−1^. Two primer pairs were selected as *A. thaliana* reference genes based on geNORM [58] analysis of expression stability and previous evaluation [59]. The two reference genes selected were a gene encoding a pentatricopeptide repeat (At5g55840; 5′-AAG ACA GTG AAG GTG CAA CCT TAC T-3′, 5′-GTT TTT GAG TTG TAT TTG TCA GAG AAA G-3′, amplicon 61 bp in length), and one encoding the mitosis-associated protein YLS8 (At5g08290; 5′-TTA CTG TTT CGG TTG TTC TCC ATT T-3′, 5′-CAC TGA ATC ATG TTC GAA GCA AGT-3′, amplicon 66 bp in length). Both amplicons were generated by imposing an initial denaturation of 95°C/3 min, followed by 40 cycles of 95°C/10 s, 60°C/30 s. The template DNA was diluted 100 fold in sterile water to an approximate concentration of 1 ng µL^−1^. The PCR efficiency of the primer pairs, as estimated from a template dilution series, was respectively 97% for VDS, 98% for YLS8 and 99% for PPR. A CFX96 real-time System driven by CFX Manager software v2.1 was used for qRT-PCR, in reactions based on SsoAdvanced™ SYBR® Green Supermix (Bio-Rad Laboratories, Hercules, CA). Each 6 µL reaction was composed of 3 µL 2× SsoAdvanced, 1 µL diluted DNA and 1 µL of each gene-specific primer (2 µM), and was replicated three times per biological sample. Primer specificity was assessed by inspection of the melting curve after cycle 40 and agarose gel electrophoresis of the amplicon. The Cq values of individual well traces were determined using the regression model implemented in the CFX Manager software. The data were analyzed using qbasePLUS software v2.3 (Biogazelle NV, Belgium) applying the following parameters: primer amplification efficiency: 100%, normalization strategy: two reference targets [60].

### Data analysis

All chemical and microbial data were checked to be normally distributed and showed homogeneity of variances before the analysis of variances (ANOVA's) were calculated using Tukey's HSD test at p≤0.001–0.05.

## Results and Discussion

### Genetic variation in *A. thaliana* for the suppression of *in vitro* growth of *V. longisporum*

**Figure 1:**
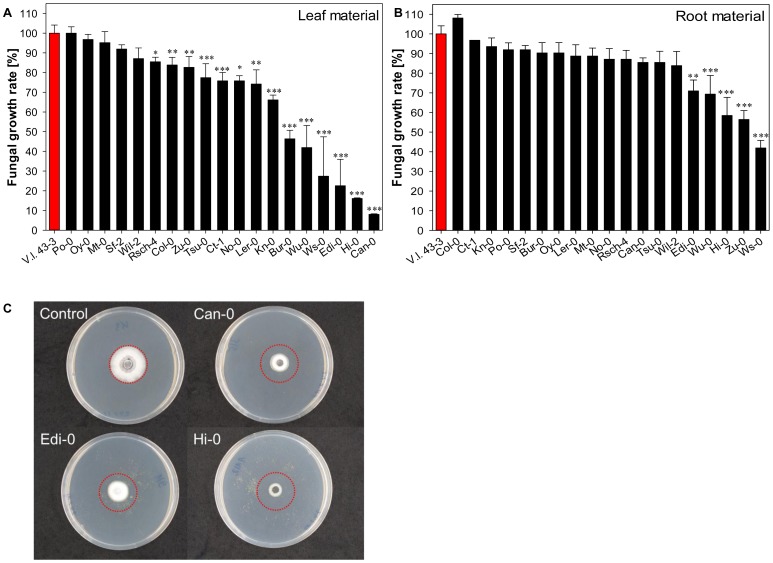
Relative growth of *Verticillium longisporum* 43-3 on PDA at 25°C for four days when exposed to volatiles emitted from 19 *Arabidopsis thaliana* accessions. Red bars represent mycelial growth in the absence of plant tissue and black bars represent (A) leaf and (B) root tissue. Data represent the mean of five replicates, and the error bar represents the standard error. Significant differences between the control mycelia and those exposed to plant material are indicated by asterisks (*: *p*<0.05, **: *p*<0.01, ***: *p*<0.001). (C) The effect on the *in vitro* growth of *V. longisporum* 43-3 of leaf volatiles emitted from the three most suppressive accessions after four days of exposure. The red circle indicates a diameter of 1.6 cm.

When *V. longisporum* 43-3 was exposed to plant material extracted from the 19 different *A. thaliana* accessions, its growth was more noticeably retarded by the presence of lyophilized leaf rather than root tissue (Fig. 1). The leaf tissue-induced reduction in growth reached 92% of the non-treated control, whereas the maximum extent of the suppression induced by the presence of root tissue was only 58%. The six accessions whose leaf tissue induced the most substantial growth reduction (>50%) were Bur-0, Can-0, Edi-0, Hi-0, Ws-0 and Wu-0. The most efficacious root tissues (reducing fungal growth by >30%) were those prepared from Edi-0, Hi-0, Ws-0, Wu-0 and Zu-0. The apparent presence of genetic variation in *A. thaliana* for the ability to suppress fungal growth mirrors equivalent variation demonstrated in other *Brassica* spp. [61], [62]. The extent of the inhibition is also comparable to that observed against *Sclerotinia sclerotiorum* [63], *Leptosphaeria maculans* [64], *Xanthomonas campestris* [41] and *V. dahliae* [65], [66].

### Genetic variation for glucosinolate composition among *A. thaliana* accessions

**Figure 2:**
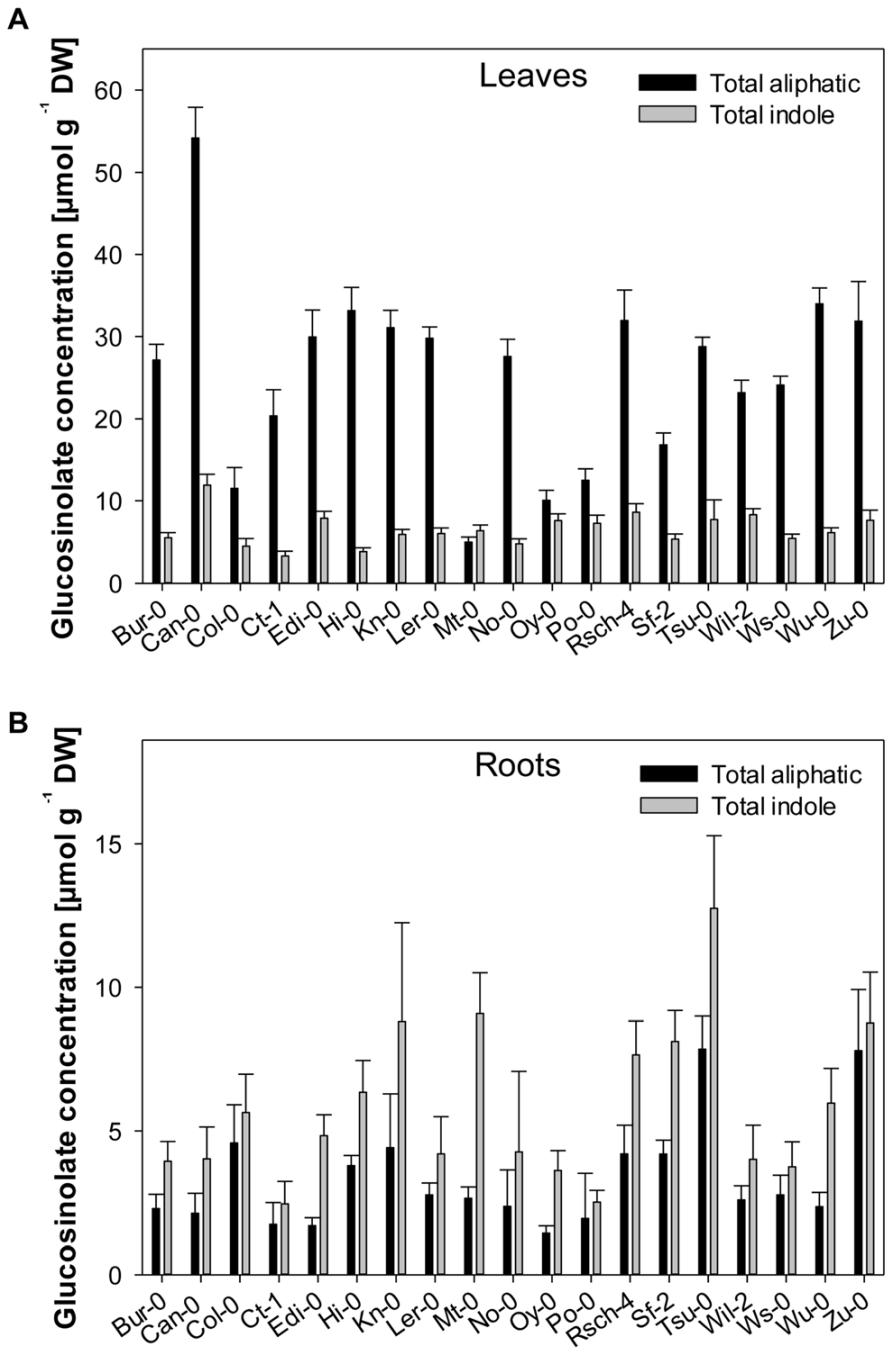
Total concentration of aliphatic and indole glucosinolates in a range of *Arabidopsis thaliana* accessions. Bars represent the cumulative total of each glucosinolate class in (A) leaf tissue and (B) root tissue, and error bars represent standard deviation.

The profile of compounds emitted by intact *A. thaliana* leaves is dominated by terpenes and various aromatic compounds, but wounding induces a shift towards that of glucosinolate hydrolysis products [67]. For this reason, our focus was to obtain the glucosinolate profiles of the 19 *A. thaliana* accessions. These profiles are known to be affected by both genetic and environmental factors [68], [69], [70], and vary between plant organs [71], [72] and over development [73]. A total of 20 distinct glucosinolates was identified and quantified, of which 16 were aliphatic (alkenyl, hydroxyalkenyl, hydroxyalkyl, thioalkyl and sulfinylalkyl glucosinolates) and four indole (Tables S1 and S2). All 20 compounds have previously been detected in *A. thaliana* [68], [74]. The aliphatic glucosinolate concentration in the leaf tissue was tenfold that in the root tissue, while the indole glucosinolates were equally represented in both tissues (Fig. 2); a similar partitioning was obtained in the Col-0 accession [71], [73]. The glucosinolate composition varied from accession to accession. While some compounds (particularly the indole glucosinolates) were present in all 19 accessions, most of the aliphatic ones were accession-specific (Tables S1 and S2). The aliphatic glucosinolate concentration in the leaf tissue varied from 5 µmol g^−1^ dry weight (DW) in Mt-0 to 54 µmol g^−1^ DW in Can-0, while the indole glucosinolate concentration lay between 3.3 µmol g^−1^ DW (Ct-1) and 12 µmol g^−1^ DW (Can-0). In root tissue, the range in aliphatic glucosinolate concentration was 1.4–7.8 µmol g^−1^ DW (for, respectively, Oy-0 and Zu-0), while that for the indole glucosinolates was higher abundant with 2.5–12.7 µmol g^−1^ DW (Ct-1 and Tsu-0) (Fig. 2). A similar range both with respect to composition and quantity has also been demonstrated in 82 different *B. rapa* cultivars [62], and in 39 [68] and 96 *A. thaliana* accessions [74].

**Figure 3:**
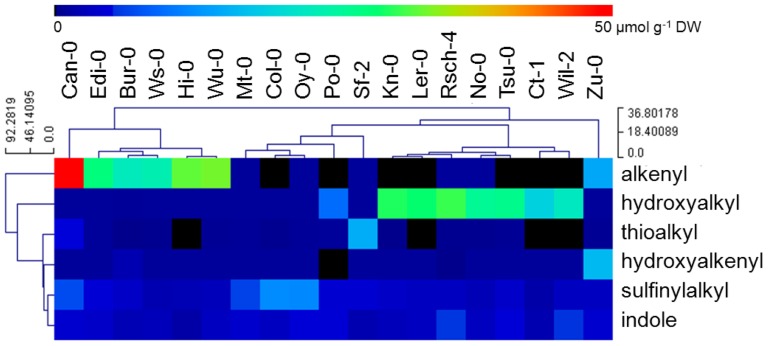
Hierarchical clustering of the glucosinolate composition of the leaf tissue of a range of *Arabidopsis thaliana* accessions. Each column represents one accession and each row the concentration of glucosinolates (µmol g^−1^ DW) using colour coding.

A hierarchical clustering was performed to group accessions on the basis of their glucosinolate profile. This analysis delivered three major clusters, the first comprising accessions Can-0, Edi-0, Bur-0, Ws-0, Hi-0 and Wu-0, which preferentially accumulated the alkenyl glucosinolates 2Prop, 3-butenyl (3But) and 4-pentenyl glucosinolate (4Pent) (with 2Prop being the most abundant); the second group featured those accumulating hydroxyalkyl glucosinolates (Kn-0, Ler-0, Rsch-4, No-0, Tsu-0, Ct-1, Wil-2), and the third those with an elevated level of methylsulfinylalkyl and indole glucosinolates (Mt-0, Col-0, Oy-0, Po-0, Sf-2) (Fig. 3). There was a correlation between an accession's ability to accumulate alkenyl glucosinolates and the suppression by its leaf tissue of *Verticillium* sp. growth. Leaf tissue prepared from Can-0 was the most effective for inhibiting fungal growth, and this accession also accumulated the most 2Prop glucosinolate; as a result, the hypothesis was that a hydrolysis product of 2Prop glucosinolate is the major agent of anti-fungal activity.

### *V. longisporum* growth is affected by the formation of 2Prop-ITC

A subset of ten Arabidopsis accessions was selected for chemical analysis based on contrasting leaf glucosinolate patterns to identify glucosinolate hydrolysis products with an inhibitory effect on Verticillium growth. Accessions included alkenyl glucosinolate accumulators with strongest antifungal effects (Bur-0, Can-0, Hi-0, and Wu-0) as well as accessions being rich in hydroxyalkenyl, hydroxyalkyl, methylthioalkyl and methylsulfinylalkyl glucosinolates that showed low antifungal activity (Kn-0, Ler-0, Po-0, Rsch-4, Wil-2, and Zu-0). A total of 20 different glucosinolate hydrolysis products was identified and quantified in lyophilized plant tissue that were either ITC (including OZT), nitriles or epithionitriles (Table 1). ITCs were the main breakdown products formed in all 10 accessions. The predominant hydrolysis product in Can-0, Hi-0, and Wu-0 was 2Prop-ITC with concentrations in the range of 9.2–19.7 µmol g^−1^ DW (Can-0 and Hi-0, respectively), whereas Bur-0 formed slightly more 3But-ITC than 2Prop-ITC. The main degradation product upon myrosinase-driven breakdown in Zu-0 was also 3But-ITC, but this accession also formed OZT in substantial amounts. Accessions Kn-0, Ler-0, Po-0, Rsch-4, and Wil-2 revealed a high level of 3-hydroxypropyl ITC (3OHP-ITC), ranging from 13–44.3 µmol g^−1^ DW for, respectively, Po-0 and Kn-0. Epithionitriles, being formed only in presence of the ESP from alkenyl or hydroxyalkenyl glucosinolates [27] were detected in hydrolysed leaf tissues of Bur-0, Can-0, Wu-0 and Zu-0, but not in those from Hi-0. Nitrile production usually is accompanied by the formation of ITC, however hydrolysed Kn-0 leaf tissue was absent of the nitrile deriving from 3OHP, although it was detected in all other hydroxyalkyl rich accessions.

**Table 1:** Breakdown products formed by hydrolysis of glucosinolates in the leaf tissue of selected *Arabidopsis thaliana* accessions.

	Bur-0	Can-0	Hi-0	Kn-0	Ler-0	Po-0	Rsch-4	Wil-2	Wu-0	Zu-0
Alkenyl hydrolysis products										
2Prop-CN	0.05±0.05	0.12±0.03	0.06±0.00	n.d.	n.d.	0.05±0.03	n.d.	n.d.	0.16±0.10	n.d.
2Prop-ITC	5.30±0.84	9.21±1.47	19.74±0.51	n.d.	n.d.	n.d.	n.d.	n.d.	16.60±6.04	2.39±0.31
2Prop-EPT	0.54±0.36	1.06±0.13	n.d.	n.d.	n.d.	n.d.	n.d.	n.d.	1.12±0.87	0.17±0.11
3But-CN	0.16±0.07	n.d.	0.16±0.02	n.d.	n.d.	n.d.	n.d.	n.d.	n.d.	0.18±0.04
3But-ITC	6.82±0.78	0.25±0.04	n.d.	n.d.	n.d.	n.d.	n.d.	n.d.	n.d.	6.44±1.00
3But-EPT	0.65±0.38	0.02±0.00	n.d.	n.d.	n.d.	n.d.	n.d.	n.d.	n.d.	0.29±0.18
4Pent-ITC	0.30±0.06	n.d.	n.d.	n.d.	n.d.	n.d.	n.d.	n.d.	n.d.	0.27±0.02
Hydroxyalkenyl hydrolysis products										
Epi2OH3But-EPT	0.04±0.05	n.d.	n.d.	n.d.	n.d.	n.d.	n.d.	n.d.	n.d.	0.49±0.26
OZT	0.19±0.23	n.d.	n.d.	n.d.	n.d.	n.d.	n.d.	n.d.	n.d.	2.63±0.35
Methylthioalkyl hydrolysis products										
3MTP-CN	n.d.	0.05±0.01	n.d.	n.d.	n.d.	n.d.	n.d.	n.d.	n.d.	n.d.
3MTP-ITC	n.d.	0.45±0.14	n.d.	n.d.	n.d.	n.d.	0.09±0.04	n.d.	n.d.	n.d.
4MTB-ITC	n.d.	0.08±0.03	n.d.	n.d.	n.d.	0.17±0.04	n.d.	n.d.	n.d.	n.d.
8MTO-CN	n.d.	0.03±0.00	n.d.	0.47±0.11	n.d.	0.02±0.02	n.d.	n.d.	n.d.	n.d.
8MTO-ITC	0.14±0.03	0.64±0.05	0.14±0.05	n.d.	0.21±0.10	0.48±0.08	0.36±0.13	0.09±0.02	0.40±0.15	0.35±0.02
Methylsulfinylalkyl hydrolysis products										
3MSOP-ITC	n.d.	0.37±0.11	n.d.	n.d.	n.d.	0.29±0.04	0.15±0.26	n.d.	n.d.	n.d.
4MSOB-ITC	n.d.	n.d.	n.d.	n.d.	n.d.	2.54±0.31	n.d.	n.d.	n.d.	n.d.
8MSOO-CN	0.22±0.10	0.32±0.02	n.d.	0.17±0.02	n.d.	0.15±0.01	0.11±0.03	n.d.	n.d.	n.d.
8MSOO-ITC	0.54±0.13	0.27±0.01	0.16±0.04	0.84±0.25	0.29±0.23	0.51±0.03	0.43±0.21	0.30±0.03	n.d.	0.17±0.11
Hydroxyalkyl hydrolysis products										
3OHP-CN	n.d.	n.d.	n.d.	n.d.	0.76±0.05	0.70±0.28	0.74±0.48	0.76±0.21	n.d.	n.d.
3OHP-ITC	n.d.	n.d.	n.d.	44.34±1.96	38.93±2.28	13.00±2.32	37.17±12.79	32.31±0.23	n.d.	n.d.

Quantities shown in µmol g^−1^ DW, derived from the mean of three batches of plants (each n = 50) and two technical replicates per sample. Errors denote standard deviation.

2Prop-CN: 3-butenenitrile, 2Prop-ITC: 2-propenyl ITC, 2Prop-EPT: 3,4-epithiobutylnitrile, 3But-CN: 4-pentenenitrile, 3But-ITC: 3-butenyl ITC, 3But-EPT: 4,5-epithiopentylnitrile, 4Pent-ITC: 4-pentenyl ITC, 2OH3But-EPT: 3-hydroxy-4,5-epithiopentylnitrile, OZT: 5-vinyl-1,3-oxazolidine-2-thione, 3MTP-CN: 4-(methylthio)butylnitrile, 3MTP-ITC: 3-(methylthio)propyl ITC, 4MTB-ITC: 4-(methylthio)butyl ITC, 8MTO-CN: 9-(methylthio)nonylnitrile, 8MTO-ITC: 8-(methylthio)octyl ITC, 3MSOP-ITC: 3-(methylsulfinyl)propyl ITC, 4MSOB-ITC: 4-(methylsulfinyl)butyl ITC, 8MSOO-CN: 9-(methylsulfinyl)nonyl ITC, 8MSOO-ITC: 8-(methylsulfinyl)octyl ITC, 3-OHP-CN: 4-hydroxybutylnitrile, 3-OHP-ITC: 3-hydroxypropyl ITC. n.d. not detected.

**Figure 4:**
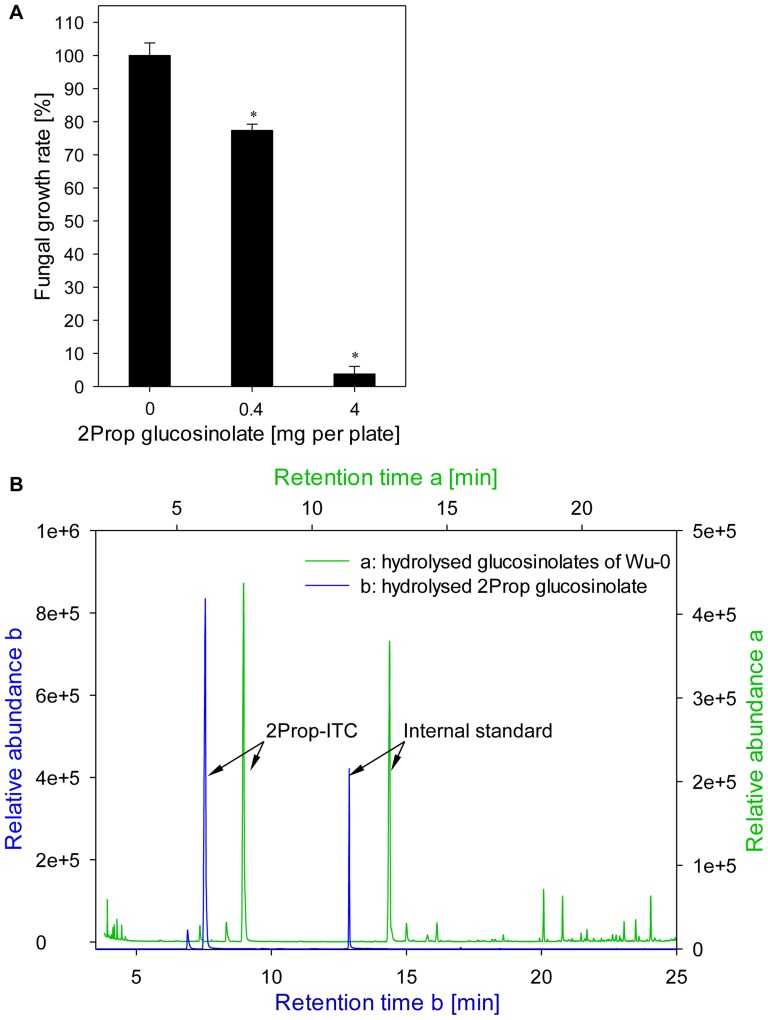
Fungitoxicity of 2Prop-ITC on growth of *Verticillium longisporum* 43-3. Effect of hydrolysed 2Prop glucosinolate on the *in vitro* growth of *V. longisporum* 43-3 was demonstrated using the biofumigation assay (A). Data represent the mean of five technical replicates per biological sample and error bars represent the standard error. Significant differences between the control mycelia and those exposed to 2Prop-ITC are indicated by asterisks (*: *p*<0.001). The formation of 2Prop-ITC through myrosinase-driven glucosinolate breakdown was verified by GC-MS analysis (B). GC-MS chromatograms display the product of hydrolysed 2Prop glucosinolate (black) in comparison to the hydrolysed fraction accession Wu-0 (green line).

Alkenyl accumulating Arabidopsis accessions, that were able to restrict growth of *V. longisporum*, formed 2Prop-ITC as main glucosinolate hydrolysis product. Therefore, the inhibitory effect of hydrolysis products of purified 2Prop glucosinolate was tested for the *V. longisporum* isolate 43-3. Fungal growth was significantly inhibited by concentrations of 0.4 and 4.0 mg 2Prop glucosinolate per plate in a dose-dependent manner (Fig. 4 A). The application of 4 mg 2Prop glucosinolate, matching the same amount in 1 g leaf material of Bur-0, resulted in a growth reduction of 97% as compared to the 54% inhibition obtained by the leaf material of the same accession. The GC-MS analysis of the hydrolysed 2Prop glucosinolate (Fig. 4 B) confirmed the formation of 2Prop-ITC as the main degradation product. After 2 h of hydrolysis time 91% of the 2Prop glucosinolate (0.4 mg level) were recovered as ITC, those concentrations declining to 63% within the next 22 h.

**Figure 5:**
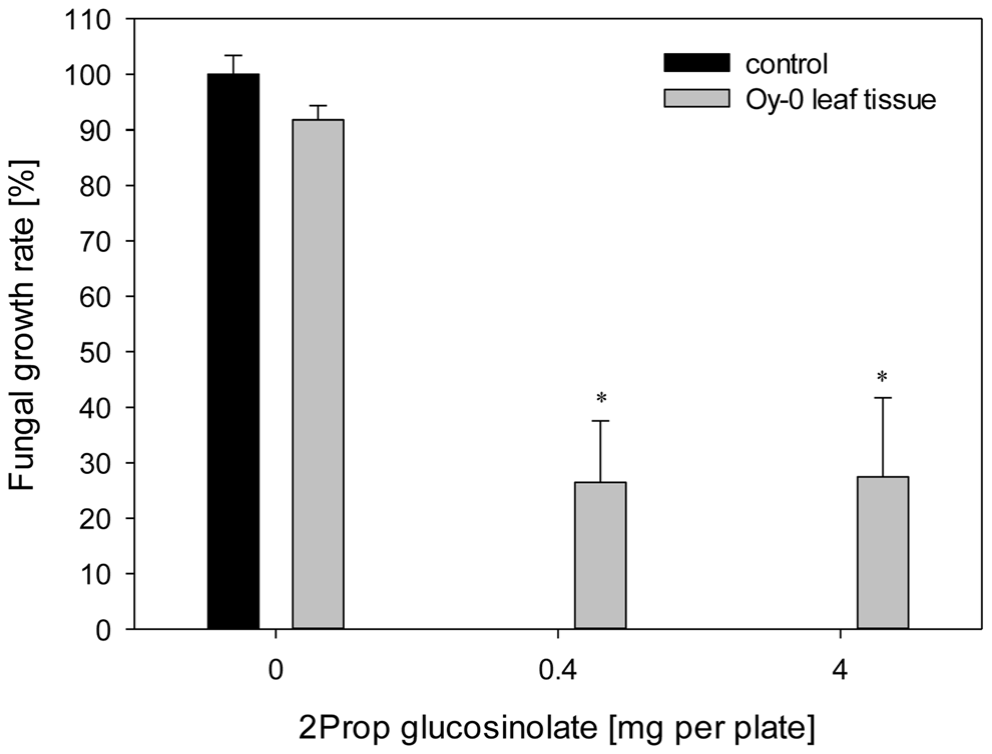
The effect of increased 2Prop glucosinolate concentrations in the low 2Prop glucosinolate accumulating *Arabidopsis thaliana* accession Oy-0 on the growth of *Verticillium longisporum* 43-3. Data represent the mean of five technical replicates per biological sample and error bars represent the standard error. Significant differences between the control mycelia and those exposed to Oy-0 volatiles spiked with 2Prop glucosinolate are indicated by asterisks (*: *p*<0.001).

Low alkenyl-accumulating *A. thaliana* accessions showed no ability to suppress fungal growth in the bioassay (see Fig. 1). The pure 2Prop glucosinolate was added to leaf material of Oy-0 in order to complement this deficiency in fungitoxicity, (Fig. 5). Fungal growth was not significantly affected by the presence of Oy-0 leaf material. However, when 0.4 or 4 mg 2Prop glucosinolate were added to the lyophilized Oy-0 leaf material, the fungal growth rate decreased to 11 and 14%, respectively, as compared to the non-treated control. This indicates that 2Prop glucosinolate greatly contributes to the growth suppression observed for alkenyl-accumulating plant accessions. Thus, present data suggest that 2Prop-ITC can provide protection against fungal pathogen infection. The abundance of 2Prop-ITC has been correlated with fungicidal activity in several *Brassica* spp. [75], [76], [77], while the exposure to purified 2Prop-ITC is strongly inhibitory over the growth of both *Fusarium oxysporum* [36], *Phymatotrichopsis omnivora* [78] and *V. dahliae* [79]. Transcriptional analysis in *A. brassicicola* points to oxidative damage and redox imbalance being the result of exposure [80].

### Differential systemic colonization by *Verticillium* ssp

**Figure 6:**
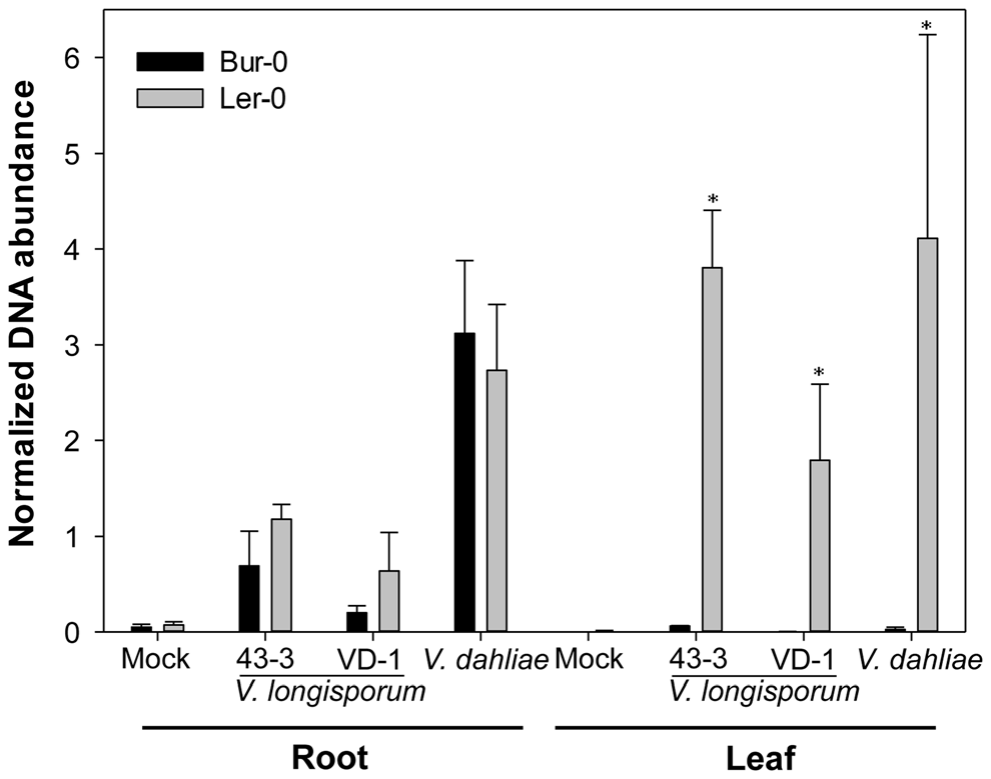
Systemic spread of *Verticillium longisporum* 43-3, VD-1 and *V. dahliae* GU060637 within the leaf and root of *Arabidopsis thaliana* accessions Ler-0 and Bur-0, as measured by qRT-PCR, five weeks after inoculation. Data represent the mean of three batches consisting of five plants each, measured in technical triplicates via qRT-PCR. Bars denote standard deviations. Significant differences between Ler-0 and Bur-0 are indicated by asterisks (*: *p*<0.001).

The accessions accumulating 2Prop glucosinolate were those whose leaf tissue most strongly inhibited the growth of *V. longisporum*. It was therefore of interest to contrast two accessions differing in their ability to accumulate 2Prop in their leaf with respect to their capacity to resist the systemic spread of the pathogen *in planta*. Since genetic mapping of resistance against *V. longisporum* infection has already been carried out in a population derived from a cross between Ler-0 and Bur-0 [20], these two accessions represented an appropriate choice of material. The extent of fungal colonization in the root and leaf tissue of the two accessions was quantified using quantitative real time PCR (qRT-PCR). The analysis was extended to a second isolate of *V. longisporum*, VD-1, and to the closely related species *V. dahliae* to test whether 2-Prop contribute to non-host resistance and inhibits growth of a range of Verticillium species. *V. dahliae* isolate accession GU060637 colonized the root system more efficiently than did either of the two *V. longisporum* isolates 43-3 and VD-1 (Fig. 6). However, there was no significant difference in the quantity of fungal DNA present in the root tissue of Ler-0 and Bur-0, suggesting that resistance is unrelated to the ability to prevent invasion. All three fungal isolates were detectable in the leaf tissue of the susceptible accession Ler-0, but not in that of Bur-0. The implication is that resistance is determined by an interaction occurring in the shoot, as also suggested in recent studies on *Verticillium* interactions with Bur-0/Ler-0 (high/low alkenyl) [20] or Ws-0/Ler-0 (high/low alkenyl) [81]. The accumulation of alkenyl glucosinolates in the leaf tissue thus seems to represent an important mechanism for plant resistance, probably acting to inhibit the systemic spread of the pathogen. Note, however, that glucosinolate hydrolysis products differ between lyophilized and fresh plant material since modifying enzymes loose activity after freeze-drying. Hence, also other factors might contribute to the suppression of fungal spread *in planta*. The interaction between the host and its various fungi and bacteria is clearly therefore a complex one.

### Concluding Remarks

We have reported here that key *A. thaliana* accessions vary with respect to their accumulation of glucosinolates in the leaf and root tissue, and that the accumulation of 2Prop glucosinolate in the leaf can explain much of the inhibitory effect of leaf tissue on the *in vitro* growth of *V. longisporum*. In order to further assess the biofumigation potential of 2Prop glucosinolate for crop protection, effectiveness should be investigated under field conditions.

## Supporting Information

Table S1 **Glucosinolates present in the leaf of a range of** ***Arabidopsis thaliana*** **accessions.** Quantities shown in µmol g^−1^ DW, derived from the mean of three batches of plants (each n = 50) and two technical replicates per sample. Errors denote standard deviation. 2Prop: 2-propenyl, 3But: 3-butenyl, 4Pent: 4-pentenyl, 2OH3But: (2R)-2-hydroxy-3-butenyl, Epi2OH3But: (2S)-2-hydroxy-3-butenyl, 3MTP: 3-(methylthio)propyl, 4MTB: 4-(methylthio)butyl, 7MTH: 7-(methylthio)heptyl, 8MTO: 8-(methylthio)octyl, 3MSOP: 3-(methylsulfinyl)propyl, 4MSOB: 4-(methylsulfinyl)butyl, 5MSOP: 5-(methylsulfinyl)pentyl, 6MSOH: 6-(methylsulfinyl)hexyl, 7MSOH: 7-(methylsulfinyl)heptyl, 8MSOO: 8-(methylsulfinyl)octyl, 3OHP: 3-hydroxypropyl, I3M: 3-indolylmethyl, 4OHI3M: 4-hydroxy-3-indolylmethyl, 1MOI3M: 1-methoxy-3-indolylmethyl, 4MOI3M: 4-methoxy-3-indolylmethyl glucosinolate. n.d. not detected.
(DOCX)

Table S2 **Glucosinolates present in the root tissue of a range of** ***Arabidopsis thaliana*** **accessions.** Quantities shown in µmol g^−1^ DW, derived from the mean of three batches of plants (each n = 50) and two technical replicates per sample. Glucosinolate abbreviations as used in Table S1. n.d.: not detected.
(DOCX)
