# Supplementary material for: Verticillium Suppression Is Associated with the Glucosinolate Composition of Arabidopsis thaliana Leaves
Source: PLoS One. 2013 Sep 5;8(9):e71877. doi: 10.1371/journal.pone.0071877 (PMC3764120; doi:10.1371/journal.pone.0071877)
Supplement: Table S1 — Glucosinolates present in the leaf of a range of Arabidopsis thaliana accessions. Quantities shown in µmol g−1 DW, derived from the mean of three batches of plants (each n = 50) and two technical replicates per sample. Errors denote standard deviation. 2Prop: 2-propenyl, 3But: 3-butenyl, 4Pent: 4-pentenyl, 2OH3But: (2R)-2-hydroxy-3-butenyl, Epi2OH3But: (2S)-2-hydroxy-3-butenyl, 3MTP: 3-(methylthio)propyl, 4MTB: 4-(methylthio)butyl, 7MTH: 7-(methylthio)heptyl, 8MTO: 8-(methylthio)octyl, 3MSOP: 3-(methylsulfinyl)propyl, 4MSOB: 4-(methylsulfinyl)butyl, 5MSOP: 5-(methylsulfinyl)pentyl, 6MSOH: 6-(methylsulfinyl)hexyl, 7MSOH: 7-(methylsulfinyl)heptyl, 8MSOO: 8-(methylsulfinyl)octyl, 3OHP: 3-hydroxypropyl, I3M: 3-indolylmethyl, 4OHI3M: 4-hydroxy-3-indolylmethyl, 1MOI3M: 1-methoxy-3-indolylmethyl, 4MOI3M: 4-methoxy-3-indolylmethyl glucosinolate. n.d. not detected. (DOCX) [file pone.0071877.s001.docx]

Table S1

|  | Bur-0 | Can-0 | Col-0 | Ct-1 | Edi-0 | Hi-0 | Kn-0 | Ler-0 | Mt-0 | No-0 | Oy-0 | Po-0 | Rsch-4 | Sf-2 | Tsu-0 | Wil-2 | Ws-0 | Wu-0 | Zu-0 |
| --- | --- | --- | --- | --- | --- | --- | --- | --- | --- | --- | --- | --- | --- | --- | --- | --- | --- | --- | --- |
|  | | |  |  |  |  |  |  |  |  |  |  |  |  |  |  |  |  |  |
| Alkenyl glucosinolates | | |  |  |  |  |  |  |  |  |  |  |  |  |  |  |  |  |  |
| 2Prop | 9.35±0.69 | 48.52±3.17 | 0.03±0.03 | 0.06±0.02 | 27.70±2.86 | 32.46±2.64 | n.d. | n.d. | n.d. | n.d. | n.d. | 0.05±0.03 | n.d. | n.d. | 0.03±0.01 | n.d. | 23.61±1.02 | 33.22±1.83 | 4.35±0.70 |
| 3But | 13.22±0.81 | 1.17±0.13 | n.d. | n.d. | 0.26±0.03 | 0.22±0.04 | n.d. | n.d. | n.d. | n.d. | n.d. | n.d. | n.d. | n.d. | 0.05±0.00 | 0.05±0.01 | 0.33±0.02 | 0.30±0.02 | 10.50±1.49 |
| 4Pent | 0.41±0.04 | n.d. | n.d. | 0.10±0.02 | 0.21±0.01 | n.d. | 0.17±0.02 | 0.18±0.03 | n.d. | n.d. | n.d. | n.d. | n.d. | n.d. | 0.11±0.00 | n.d. | n.d. | n.d. | n.d. |
|  | | | |  |  |  |  |  |  |  |  |  |  |  |  |  |  |  |  |
| Hydroxyalkenyl glucosinolates | | | |  |  |  |  |  |  |  |  |  |  |  |  |  |  |  |  |
| 2OH3But | 0.59±0.05 | n.d. | n.d. | n.d. | n.d. | n.d. | n.d. | n.d. | n.d. | n.d. | n.d. | 0.06±0.01 | n.d. | n.d. | n.d. | n.d. | n.d. | n.d. | 3.93±0.69 |
| Epi2OH3But | 1.71±0.12 | n.d. | n.d. | n.d. | n.d. | n.d. | n.d. | n.d. | n.d. | n.d. | n.d. | n.d. | 0.20±0.03 | n.d. | n.d. | n.d. | n.d. | n.d. | 12.62±1.86 |
|  | | |  |  |  |  |  |  |  |  |  |  |  |  |  |  |  |  |  |
| Methylthioalkyl glucosinolates | | | |  |  |  |  |  |  |  |  |  |  |  |  |  |  |  |  |
| 3MTP | 0.16±0.02 | 2.27±0.09 | n.d. | n.d. | 0.67±0.09 | n.d. | n.d. | n.d. | n.d. | n.d. | 0.15±0.02 | n.d. | n.d. | 2.43±0.21 | 0.06±0.01 | n.d. | n.d. | n.d. | n.d. |
| 4MTB | n.d. | 0.45±0.06 | 0.42±0.15 | n.d. | 0.08±0.02 | n.d. | n.d. | n.d. | 0.61±0.07 | n.d. | n.d. | 0.46±0.05 | n.d. | 12.10±0.95 | 0.09±0.01 | n.d. | n.d. | n.d. | n.d. |
| 7MTH | 0.11±0.01 | 0.47±0.14 | 0.05±0.02 | 0.02±0.00 | 0.09±0.03 | 0.02±0.01 | 0.05±0.01 | 0.04±0.01 | 0.05±0.00 | 0.05±0.01 | 0.08±0.01 | 0.11±0.02 | 0.09±0.02 | 0.31±0.06 | 0.09±0.00 | 0.01±0.00 | 0.08±0.01 | 0.10±0.01 | 0.25±0.04 |
| 8MTO | 0.12±0.01 | 1.87±0.22 | 0.07±0.02 | 0.08±0.02 | 0.32±0.09 | 0.05±0.02 | 0.18±0.06 | 0.09±0.02 | 0.25±0.03 | 0.39±0.12 | 0.58±0.07 | 0.52±0.16 | 0.49±0.13 | 0.63±0.05 | 0.41±0.02 | 0.05±0.02 | 0.49±0.07 | 0.49±0.12 | 0.39±0.06 |
|  | | |  |  |  |  |  |  |  |  |  |  |  |  |  |  |  |  |  |
| Methylsulfinylalkyl glucosinolates | | | |  |  |  |  |  |  |  |  |  |  |  |  |  |  |  |  |
| 3MSOP | n.d. | 1.04±0.16 | 1.43±0.09 | 0.72±0.29 | 0.59±0.19 | 0.22±0.09 | 0.36±0.04 | 0.28±0.03 | 0.50±0.10 | 1.44±0.12 | 9.57±1.14 | 0.38±0.06 | 0.43±0.03 | 0.39±0.07 | 1.02±0.11 | 0.11±0.02 | 0.07±0.01 | 0.25±0.06 | n.d. |
| 4MSOB | n.d. | n.d. | 9.06±2.13 | 0.09±0.02 | n.d. | n.d. | 0.25±0.08 | n.d. | 3.65±0.41 | n.d. | 0.15±0.02 | 1.74±0.14 | n.d. | 1.60±0.14 | 0.24±0.02 | 0.10±0.10 | n.d. | n.d. | n.d. |
| 5MSOP | n.d. | n.d. | 0.28±0.07 | n.d. | 0.08±0.03 | n.d. | n.d. | n.d. | n.d. | n.d. | n.d. | 0.02±0.02 | n.d. | 0.01±0.00 | n.d. | n.d. | n.d. | n.d. | n.d. |
| 6MSOH | n.d. | n.d. | n.d. | n.d. | 0.05±0.01 | n.d. | n.d. | n.d. | n.d. | n.d. | n.d. | n.d. | n.d. | 0.04±0.01 | n.d. | n.d. | n.d. | n.d. | n.d. |
| 7MSOH | 0.72±0.09 | 0.76±0.09 | 0.30±0.09 | 0.11±0.02 | 0.30±0.04 | 0.26±0.05 | 0.24±0.02 | 0.24±0.03 | 0.18±0.02 | n.d. | 0.09±0.02 | 0.13±0.03 | 0.19±0.03 | 0.27±0.06 | 0.19±0.01 | 0.21±0.02 | 0.11±0.01 | 0.21±0.02 | 0.44±0.09 |
| 8MSOO | 2.95±0.31 | 5.27±0.42 | 1.19±0.30 | 0.90±0.16 | 3.64±0.47 | 1.99±0.27 | 2.84±0.30 | 2.68±0.16 | 2.05±0.13 | 1.12±0.09 | 2.15±0.30 | 2.06±0.22 | 2.58±0.34 | 2.19±0.22 | 2.46±0.15 | 2.89±0.33 | 1.99±0.20 | 2.33±0.21 | 2.89±0.45 |
|  | | | |  |  |  |  |  |  |  |  |  |  |  |  |  |  |  |  |
| Hydroxyalkyl glucosinolates | | | |  |  |  |  |  |  |  |  |  |  |  |  |  |  |  |  |
| 3OHP | 0.98±0.10 | n.d. | n.d. | 19.28±2.82 | n.d. | 0 n.d. | 30.05±1.97 | 29.09±1.30 | n.d. | 26.13±1.94 | n.d. | 9.61±1.04 | 31.02±3.61 | n.d. | 27.00±0.92 | 22.71±1.40 | n.d. | n.d. | n.d. |
|  | | |  |  |  |  |  |  |  |  |  |  |  |  |  |  |  |  |  |
| Indole glucosinolates | | |  |  |  |  |  |  |  |  |  |  |  |  |  |  |  |  |  |
| I3M | 1.50±0.17 | 2.55±0.34 | 2.50±0.39 | 1.81±0.31 | 2.95±0.15 | 1.35±0.12 | 2.04±0.17 | 2.43±0.32 | 3.22±0.46 | 2.36±0.25 | 3.57±0.28 | 3.50±0.37 | 4.36±0.32 | 1.49±0.21 | 3.86±2.16 | 3.84±0.24 | 2.07±0.15 | 2.47±0.18 | 3.11±0.52 |
| 4OHI3M | 0.04±0.01 | n.d. | n.d. | n.d. | n.d. | n.d. | n.d. | n.d. | 0.03±0.01 | 0.03±0.01 | 0.10±0.02 | 0.05±0.02 | 0.10±0.03 | n.d. | 0.02±0.00 | n.d. | n.d. | n.d. | 0.03±0.01 |
| 1MOI3M | 0.28±0.04 | 0.35±0.05 | 0.22±0.11 | 0.33±0.06 | 0.45±0.03 | 0.23±0.04 | 0.48±0.07 | 0.53±0.06 | 0.16±0.02 | 0.18±0.06 | 0.25±0.04 | 0.16±0.04 | 0.17±0.09 | 0.15±0.02 | 0.23±0.03 | 0.75±0.07 | 0.32±0.04 | n.d. | 0.31±0.04 |
| 4MOI3M | 0.53±0.07 | 1.40±0.15 | 0.44±0.10 | 0.14±0.03 | 0.43±0.06 | 0.15±0.03 | 0.32±0.02 | 0.24±0.12 | 0.61±0.07 | 0.66±0.07 | 0.95±0.13 | 0.90±0.18 | 0.91±0.13 | 0.56±0.06 | 0.67±0.04 | 0.75±0.09 | 0.48±0.04 | 0.72±0.06 | 0.69±0.13 |
