# Supplementary material for: Verticillium Suppression Is Associated with the Glucosinolate Composition of Arabidopsis thaliana Leaves
Source: PLoS One. 2013 Sep 5;8(9):e71877. doi: 10.1371/journal.pone.0071877 (PMC3764120; doi:10.1371/journal.pone.0071877)
Supplement: Table S2 — Glucosinolates present in the root tissue of a range of Arabidopsis thaliana accessions. Quantities shown in µmol g−1 DW, derived from the mean of three batches of plants (each n = 50) and two technical replicates per sample. Glucosinolate abbreviations as used in Table S1. n.d.: not detected. (DOCX) [file pone.0071877.s002.docx]

Table S2

|  | Bur-0 | Can-0 | Col-0 | Ct-1 | Edi-0 | Hi-0 | Kn-0 | Ler-0 | Mt-0 | No-0 | Oy-0 | Po-0 | Rsch-4 | Sf-2 | Tsu-0 | Wil-2 | Ws-0 | Wu-0 | Zu-0 |
| --- | --- | --- | --- | --- | --- | --- | --- | --- | --- | --- | --- | --- | --- | --- | --- | --- | --- | --- | --- |
|  | | |  |  |  |  |  |  |  |  |  |  |  |  |  |  |  |  |  |
| Alkenyl glucosinolates | | |  |  |  |  |  |  |  |  |  |  |  |  |  |  |  |  |  |
| 2Prop | 0.67±0.15 | 2.04±0.65 | n.d. | n.d. | 1.69±0.27 | 3.5±0.31 | n.d. | n.d. | n.d. | n.d. | n.d. | n.d. | n.d. | n.d. | n.d. | 0.03±0 | 2.56±0.53 | 2.08±0.41 | 1.07±0.3 |
| 3But | 0.83±0.14 | n.d. | n.d. | n.d. | n.d. | n.d. | n.d. | n.d. | n.d. | n.d. | n.d. | n.d. | n.d. | n.d. | n.d. | n.d. | 0.06±0.04 | n.d. | 2.04±0.6 |
| 4Pent | 0.04±0.01 | n.d. | n.d. | n.d. | n.d. | n.d. | n.d. | n.d. | n.d. | n.d. | n.d. | n.d. | n.d. | n.d. | n.d. | n.d. | n.d. | n.d. | n.d. |
|  | | | |  |  |  |  |  |  |  |  |  |  |  |  |  |  |  |  |
| Hydroxyalkenyl glucosinolates | | | |  |  |  |  |  |  |  |  |  |  |  |  |  |  |  |  |
| 2OH3But | 0.13±0.03 | n.d. | n.d. | n.d. | n.d. | n.d. | n.d. | n.d. | n.d. | n.d. | n.d. | n.d. | n.d. | n.d. | n.d. | n.d. | n.d. | n.d. | 1.13±0.27 |
| Epi2OH3But | 0.24±0.07 | n.d. | n.d. | n.d. | n.d. | n.d. | n.d. | n.d. | n.d. | n.d. | n.d. | n.d. | n.d. | n.d. | n.d. | n.d. | n.d. | n.d. | 2.94±0.78 |
|  | | |  |  |  |  |  |  |  |  |  |  |  |  |  |  |  |  |  |
| Methylthioalkyl glucosinolates | | | |  |  |  |  |  |  |  |  |  |  |  |  |  |  |  |  |
| 3MTP | n.d. | n.d. | n.d. | n.d. | n.d. | n.d. | n.d. | n.d. | n.d. | n.d. | n.d. | n.d. | n.d. | 0.46±0.06 | n.d. | n.d. | n.d. | n.d. | n.d. |
| 4MTB | n.d. | n.d. | 0.16±0.05 | n.d. | n.d. | n.d. | n.d. | 0.11±0.03 | 0.24±0.04 | n.d. | n.d. | 0.06±0.05 | n.d. | 2.69±0.21 | n.d. | n.d. | n.d. | n.d. | n.d. |
| 7MTH | 0.07±0.02 | 0.03±0.01 | 0.08±0.02 | 0.01±0 | 0.07±0.01 | 0.13±0.03 | 0.11±0.04 | 0.05±0.01 | 0.3±0.03 | 0.08±0.06 | 0.07±0.01 | 0.07±0.01 | 0.15±0.03 | 0.3±0.06 | 0.19±0.03 | 0.06±0.02 | 0.06±0.01 | 0.18±0.06 | 0.35±0.07 |
| 8MTO |  | 0.19±0.09 | 0.34±0.08 | 0.12±0.04 | 0.88±0.21 | 0.98±0.21 | 0.92±0.39 | 0.48±0.21 | 2.37±0.34 | 1.04±0.76 | 0.88±0.15 | 0.58±0.09 | 1.54±0.23 | 1.62±0.28 | 1.92±0.32 | 0.64±0.16 | 0.91±0.2 | 2.04±0.48 | 1.51±0.37 |
|  | | |  |  |  |  |  |  |  |  |  |  |  |  |  |  |  |  |  |
| Methylsulfinylalkyl glucosinolates | | | |  |  |  |  |  |  |  |  |  |  |  |  |  |  |  |  |
| 3MSOP | n.d. | n.d. | 0.42±0.14 | 0.12±0.1 | n.d. | 0.08±0.02 | 0.12±0.06 | 0.05±0.02 | 0.17±0.02 | 0.15±0.06 | 1.29±0.22 | 0.06±0.04 | 0.09±0.03 | 0.16±0.03 | 0.41±0.1 | n.d. | 0.08±0.06 | 0.09±0.03 | 0.09±0.02 |
| 4MSOB | n.d. | n.d. | 3.63±1.04 | n.d. | n.d. | n.d. | 0.05±0.02 | n.d. | 1.87±0.26 | n.d. | n.d. | 0.27±0.16 | n.d. | 0.49±0.12 | 0.06±0.05 | n.d. | n.d. | n.d. | n.d. |
| 5MSOP | n.d. | n.d. | 0.13±0.03 | n.d. | n.d. | n.d. | n.d. | n.d. | n.d. | n.d. | n.d. | n.d. | n.d. | 0.02±0 | n.d. | n.d. | n.d. | n.d. | n.d. |
| 6MSOH | n.d. | n.d. | n.d. | n.d. | n.d. | n.d. | n.d. | n.d. | n.d. | n.d. | n.d. | n.d. | n.d. | n.d. | n.d. | n.d. | n.d. | n.d. | n.d. |
| 7MSOH | 0.35±0.07 | n.d. | 0.19±0.05 | 0.07±0.01 | n.d. | 0.19±0.03 | 0.23±0.08 | n.d. | 0.17±0.03 | n.d. | 0.04±0.01 | 0.1±0.07 | 0.12±0.03 | 0.3±0.05 | 0.19±0.03 | 0.05±0.02 | 0.08±0.05 | 0.12±0.02 | 0.35±0.07 |
| 8MSOO | 1.29±0.17 | 1.17±0.25 | 1±0.34 | 0.53±0.15 | 1.45±0.18 | 2.26±0.45 | 3.57±1.31 | 2.02±0.61 | 1.93±0.3 | 0.75±0.48 | 0.77±0.18 | 0.58±0.08 | 1.61±0.32 | 2.1±0.25 | 2.33±0.43 | 0.83±0.24 | 0.94±0.23 | 1.79±0.18 | 2.55±0.44 |
|  | | | |  |  |  |  |  |  |  |  |  |  |  |  |  |  |  |  |
| Hydroxyalkyl glucosinolates | | | |  |  |  |  |  |  |  |  |  |  |  |  |  |  |  |  |
| 3OHP | n.d. | n.d. | n.d. | 1.56±0.65 | n.d. | n.d. | 4±1.72 | 2.59±0.36 | n.d. | 2.08±1.11 | n.d. | 1.34±1.1 | 3.68±0.9 | n.d. | 7.01±0.92 | 2.52±0.47 | n.d. | n.d. | n.d. |
|  | | |  |  |  |  |  |  |  |  |  |  |  |  |  |  |  |  |  |
| Indole glucosinolates | | |  |  |  |  |  |  |  |  |  |  |  |  |  |  |  |  |  |
| I3M | 0.36±0.08 | 0.34±0.11 | 0.71±0.26 | 0.33±0.09 | 0.47±0.07 | 0.63±0.1 | 0.89±0.32 | 0.31±0.08 | 1.02±0.16 | 0.58±0.36 | 0.63±0.13 | 0.35±0.07 | 0.99±0.13 | 0.82±0.06 | 1.92±0.4 | 0.46±0.11 | 0.36±0.08 | 1.13±0.18 | 0.53±0.12 |
| 4OHI3M | 0.02±0.01 | 0.09±0.05 | 0.05±0.02 | n.d. | 0.01±0 | 0.02±0 | 0.03±0.01 | 0.02±0.01 | 0.2±0.04 | 0.15±0.09 | 0.11±0.03 | 0.12±0.16 | 0.31±0.04 | 0.09±0.01 | 0.18±0.05 | n.d. | n.d. | 0.08±0.04 | 0.19±0.08 |
| 1MOI3M | 1.68±0.27 | 1.85±0.48 | 3.2±0.59 | 1.24±0.4 | 1.78±0.22 | 2.14±0.27 | 2.95±1.22 | 1.1±0.29 | 2.74±0.46 | 1.25±0.77 | 0.81±0.14 | 0.47±0.08 | 2.42±0.36 | 2.17±0.28 | 4.48±0.99 | 1.64±0.56 | 1.23±0.27 | n.d. | 3.11±0.61 |
| 4MOI3M | 0.33±0.08 | 0.45±0.16 | 0.31±0.07 | 0.23±0.1 | 0.19±0.05 | 0.2±0.06 | 0.4±0.15 | 0.23±0.09 | 0.74±0.13 | 0.57±0.38 | 0.47±0.08 | 0.46±0.08 | 0.94±0.13 | 1.11±0.14 | 1.91±0.35 | 0.39±0.1 | 0.26±0.07 | 0.83±0.3 | 0.73±0.14 |
